# Multiple health complaints in preadolescence and hospital contacts during adolescence: a prospective cohort study

**DOI:** 10.1038/s41598-022-11167-y

**Published:** 2022-05-06

**Authors:** Martin Bernstorff, Charlotte Ulrikka Rask, Dorte Rytter, Stefan Nygaard Hansen, Bodil Hammer Bech

**Affiliations:** 1 Research Unit for Epidemiology, Department of Public Health, Aarhus University, Bartholins Allé 2, 8000 Aarhus C, Denmark; 2 Department of Child and Adolescent Psychiatry, Research Unit, Aarhus University Hospital, Aarhus, Denmark; 3 Department of Clinical Medicine, Aarhus University, Aarhus, Denmark; 4 Research Unit for Biostatistics, Department of Public Health, Aarhus University, Aarhus, Denmark

**Keywords:** Psychiatric disorders, Health care, Disease prevention, Health policy, Health services, Paediatrics, Public health

## Abstract

Multiple health complaints (MHC) is increasing among preadolescents in many countries, but their prognostic effect for individual thriving or societal resource use is scarcely studied. This makes interpreting the significance of this increase challenging. We contribute by examining whether MHC in preadolescence predicts hospital contacts in adolescence by doing a nation-wide population-based cohort-study following preadolescents from the Danish National Birth-Cohort from 2010 to 2018. 96,382 children were invited at age 11. Responses to a modified version of the Health Behaviour in School Children Symptom Checklist (headache, dizziness, stomachache, irritability, feeling nervous, difficulty in getting to sleep and feeling low) was dichotomized into MHC (≥ 2 concurrent symptoms, each with a frequency of at least weekly, yes/no). Hospital contacts were derived from Danish registers from the date of answering the questionnaire to December 31st 2018. Negative binomial regression estimated incidence rate ratios (IRRs) comparing children with MHC to children without. Analyses were further broken down by hospital sector (psychiatric/somatic) and contact type (in-patient/out-patient/emergency room). 47,365 (49.1%) responded. Mean age was 11.2 years, 52% girls. 10.3% of responders reported MHC. For hospital contacts, the unadjusted IRR was 1.74 [95% CI 1.65, 1.83]. Results were robust to adjustment for sociodemographic variables and somatic/psychiatric morbidity diagnosed before baseline, IRR 1.62 [95% CI 1.54–1.71]. In conclusion, MHC in preadolescents are prognostic of hospital contacts. This shows that we cannot ignore MHC, and to prevent potentially unhelpful healthcare use, we must act. Future research should focus on the underlying causes of MHC to understand which changes will be most helpful and thus how to act.

## Introduction

Across the world, many adolescents experience symptoms daily. Symptoms such as headache, feeling nervous or trouble sleeping are very prevalent but exhibit large variation between countries. For example, estimates for “headache more than once a week” among adolescents range between 7 and 34% within Europe alone^1^. One might describe these as non-specific symptoms, as in the majority of adolescents they are not attributed to a well-defined physical or psychiatric disorder.

Studies have found non-specific symptoms to be associated with primary care use^2^, medication redemption^3^ and long-term social welfare use^4^, and some of these associations persist well into adulthood^5^. However, previous studies have used heterogeneous measures to assess these symptoms which makes the comparison of study results challenging.

One such measure, the Health Behaviour in School Children Symptom Checklist (HBSC-SCL), has been employed by the World Health Organization (WHO) in over 40 countries for more than 30 years^1,6^. It covers eight symptoms, including headache, nervousness, stomach-ache and irritability. The WHO reports the response to these items as a yes/no outcome, Multiple Health Complaints (MHC), if a participant reports two or more symptoms with a frequency of at least weekly. This makes sense as such symptoms have been shown to correlate^7^, and as the number of symptoms is also associated with impairment^8^ and healthcare use^9^.

There is still large uncertainty regarding what MHC and the underlying symptoms signify. Qualitatively, children and adolescents interpret them as being due to a myriad of factors, ranging from bodily discomfort to severe mental illness^10^. In the general population, this heterogeneity makes it premature to commit to a given causal model.

While there remains uncertainty about the cause(s), reported MHC is showing small increases internationally^11^ but marked increases in some regions. Among Scandinavian adolescents, the prevalence of MHC has increased almost 50% from 1994 to 2010^11^. Given the associations between other measures of non-specific symptoms and adverse outcomes outlined above, this is worrying.

However, whether the increase in MHC is a red flag is determined not only by how they are interpreted by those experiencing them, but also by whether they are prognostic of healthcare use and other adverse outcomes. If they are not, they are somewhat less urgent.

While the WHO has been aggregating multiple symptoms into two categories, MHC (yes/no), for over 30 years, the prognostic value is still poorly studied. To the best of our knowledge, only one study has been performed, where we showed that MHC was prognostic of increased redemption of prescription medication^3^.

This leaves a gap in the literature, as the consequence of increasing MHC levels in the population is unclear. Furthermore, to the best of our knowledge, the prognostic effect of non-specific symptoms on secondary healthcare use has not been studied at all with any measure.

The present study aims to fill these gaps by examining whether MHC is prognostic of hospital use during adolescence using a prospective design and, if so, whether this prognostic ability is independent of other factors that affect hospital contact.

Lastly, since non-specific symptoms tend to correlate, we hypothesise that a subgroup with a high symptom load is responsible for a sizeable amount of healthcare contacts. We examine this question by investigating the dose–response relationship between symptom load and the number of hospital contacts.

## Methods

### Population and design

The study was based on follow-up data from the Danish National Birth Cohort, described in detail elsewhere by the cohort’s initiators^12^. Briefly, from 1996 to 2002, the Danish National Birth Cohort recruited 100,415 pregnant women at their first pregnancy visit from the entirety of Denmark.

For the 11-year follow-up, 96,382 live-born children were sent a questionnaire including the HBSC-SCL. A single response per child was collected between 2010 and 2013. Of these, 47,365 (49%) children responded to all the relevant questions for the current study. In total, 1522 children responded to the questionnaire more than once by mistake, for whom we kept the latest response. The index date was defined as the date of filling out the 11-year questionnaire for responders and the 11th birthday for non-responders. Invitees were followed until 31st of December 2018 or death, whichever came first. Time at risk was the entire time spent during follow-up, subtracting time spent emigrated or admitted to a hospital.

To obtain information on both somatic and psychiatric hospital contacts, this information was linked to the Danish National Patient Registry^13^ via the personal identification number unique to each Dane.

### Predictor

In the present study, non-specific symptoms are estimated based on the child’s response to a modified version of the HBSC-SCL. It consists of 7 symptoms: headache, dizziness, stomachache, irritability, feeling nervous, difficulty in getting to sleep and feeling low. Each symptom is rated by its frequency during the last 6 months (nearly every day, more than once a week, about every week, nearly every month, rarely or never).

The initiators of the Danish National Birth Cohort collected one additional response category (“more than once a month”) for the frequency of each symptom. To maintain comparability, and since previous studies have shown that the HBSC-SCL has poor discriminating power around “more than once a month”^14,15^, we merged these categories. Furthermore, the initiators of the Danish National Birth Cohort did not include the backache item.

The HBSC-SCL has, in general, appropriate psychometric properties. Responses tend to be internally consistent, with Cronbach’s α ranging from 0.73 to 0.82^6,14–16^ (0.78 in the present study). It correlates negatively with scales of well-being^6,16^ and correlates positively with scales of depression^16^. Based on its wording, the HBSC-SCL seems to fit two dimensions: symptoms classically attributed to somatic disease (headache, stomachache and dizziness) and those classically attributed to psychiatric disease (irritability, feeling nervous, difficulty in getting to sleep and feeling low). However, sufficient statistical model fit has been demonstrated both for a one^6,15–17^- and two-dimensional^6,16–18^ model. Lastly, the HBSC-SCL has been validated for use in the general population and our age-group^17,19^.

For our primary analyses, the predictor was the WHO’s definition of MHC^1^: ≥ 2 concurrent symptoms, each with a frequency of at least weekly (yes/no).

For secondary analyses, the predictor was the sum score. Each symptom was weighted by its frequency (0–4, with 0 being “rarely or never”), and this weight was summed for all symptoms giving a range of 0–28.

### Outcome

The main outcome was the total number of hospital contacts, including emergency room, in- and outpatient visits to any hospital department. A contact was defined as a new contact on a day with no other contacts. If multiple contacts were initiated on the same day, only the first contact in the following order was counted: in-patient, emergency room, out-patient. For sub-analyses, contacts were separated into psychiatric and somatic (all contacts not to a psychiatric department) by which type of department was reimbursed for the contact. Furthermore, contacts were broken down by in-patient, out-patient and emergency room contacts.

### Co-variates

To examine whether there was an independent prognostic benefit of MHC we sought to adjust for other factors that are associated with healthcare seeking. Specifically, we adjusted for known child morbidity and child and parental sociodemographics^20^ at baseline.

#### Child variables

The child's sex and date of birth were collected from the Civil Registration System^21^. Somatic and psychiatric morbidity in the child at baseline was inferred from the Danish National Patient Registry and the Danish Register of Medicinal Product Statistics^22^. Specifically, all hospital visits prior to the index date were examined for each child. A child was counted as “having” a given diagnosis if any visit had that diagnosis as its main diagnosis. These diagnoses were aggregated into somatic morbidity (0–9) based on the number of the following diagnoses they had at baseline: asthma, arthritis, diabetes, disease in the nerves, muscles etc., epilepsy, heart disease, intestinal disease, kidney disease or serious vision or hearing disability (for diagnostic codes, see Supplementary Table S1). Children were categorized as having psychiatric morbidity (yes/no) if they had any hospital visit with a main diagnosis from the ICD-10 Mental and Behavioural Disorders subchapter (F).

The above scoring system has been used in previous studies^2^ and appears valid by including the most common chronic diseases among children. However, it has not been studied rigorously. As an alternative, we inferred the Charlson Comorbidity Index (CCI) from the diagnostic codes in the Danish National Patient Registry. It is a well-known measure of somatic morbidity which has been extensively validated in adults with severe disease^23^. In children, it has been shown to be predictive of healthcare costs^24^, but it explains a low amount of variance^24^. This is likely due to not including many of the common paediatric diseases from the above-mentioned scale (e.g. asthma).

#### Parental variables

Parity at birth of the child^25^, maternal education^26^ and parental cohabitation^27^ have all been shown to be associated to healthcare use. We included them as adjustment variables to see if MHC retained potential independent prognostic value. Information on these variables was obtained from Statistics Denmark^28^.

### Statistical analyses

All models were defined prior to conducting the study. Time at risk were the entire time spent from index date to the 31st of December, subtracting time spent emigrated or admitted to a hospital. In case of death, children were censored at this time. Poisson regression, negative binomial regression and zero-inflated negative binomial regression were compared by the Akaike Information Criterion^29^, with negative binomial regression providing the best fit. We used cluster robust variances at the maternal level to adjust for correlation between siblings^30^.

Model 1 examined the unadjusted association between MHC and total number of hospital contacts by negative binomial regression to estimate incidence rate ratios (IRR) and 95% confidence intervals (95% CI).

Model 2 adjusted model 1 for age at index date and sex.

Model 3 further adjusted model 2 for the number of somatic morbidities (0–9) and psychiatric morbidity (yes/no) at index date. Model 3b was the same as model 3, but used the Charlson Comorbidity Index for somatic morbidity.

Model 3 was further supplemented by model 4. It adjusted for all variables in model 3, maternal parity at the birth of the child (1–5), the last completed education by the mother on the International Standard Classification of Education 97^31^ (1–8) and parental cohabitation (yes/no) at the child’s index date.

We also examined whether the results were stable over time by plotting the incidence rate ratios by years from index date.

To examine the relationship between the HBSC-SCL sum-score and total number of hospital contacts, all the above regression models were fit with the sum-score as the predictor. The sum-score entered the regression models via a restricted cubic spline with 5 knots at the points recommended by Harrell^32^.

Since MHC is a dichotomization of a discrete scale, there is underlying variance. To visualise this, we plotted histograms of HBSC-SCL sum-score by MHC-category.

The linearity assumption was visually inspected for the only continuous variable (age at index date) and found to be appropriate. To comply with Danish data-protection legislation, we report pseudo-medians and pseudo-interquartile intervals, calculated by ordering all observations and averaging the 5 values closest to the relevant percentile. For exposure, we only completed regressions on responders, who by definition completed the relevant questionnaires and thus had no missing data. For the outcome, the lack of a record reflected the lack of a hospital visit, not missing data. And for our covariates, the dataset was complete. As such, we handled no missing data.

Analyses were all completed in Stata 15.0 (StataCorp LP, TX, USA).

### Ethics

The study was approved by the Danish Data Protection Agency under the Aarhus University comment agreement (j. number 2015-57-0002) and Aarhus University j. number 2016-051-000001, sequential number 528. According to guidelines from the Committee on Health Research Ethics in the Central Denmark Region, no ethical approval was required for this study. The Danish National Birth Cohort was approved by the Committee on Health Research Ethics in the Capital Region of Denmark j. number (KF) 01-471/94 and by the Danish Data Protection Agency under the common agreement for Statens Serum Institut, j. number 2015-57-0102. Written informed consents to use the self-reported information with linkage to register information was obtained from all mothers on behalf of themselves and their child and all methods were carried out in accordance with relevant guidelines and regulations including the General Data Protection Regulation (GDPR).

### Additional Information

The authors have no conflicts of interest to disclose, and funders played no role in study design, collection, analysis or interpretation of data, the writing of the report or the decision to submit the paper for publication.

### Role of funding sources

Funders had no role in designing or reporting the present study.

## Results

### Description of study population

A total of 47,365 children responded, 52.3% of which were girls (Table 1). The mean age of responders at index date was 11.2 years and they were followed for an average of 6.9 years. Preadolescents responding to the 11-year follow-up were broadly similar to non-responders on year of birth, maternal parity at the birth of the child, and whether their parents were cohabiting. However, some differences were found, with responders’ mothers having notably higher education, responders having very slightly fewer contacts to hospital, and responders having less somatic and psychiatric morbidity prior to the index date. Marked differences were found on sex, with responders being more likely to be girls (52.3% vs. 45.3%) (Table 1).

**Table 1:** Characteristics of responders and non-responders at the 11-year follow-up of the Danish National Birth Cohort.

	Non-responders	Responders	Total
Overall, n	49,017 (50.9)	47,365 (49.1)	96,382 (100.0)
Sex (Girls), n (%)	22,229 (45.3)	24,750 (52.3)	46,979 (48.7)
Year of birth, mean (sd)	2000.3 (1.5)	2000.2 (1.4)	2000.2 (1.4)
Somatic morbidity^a^ (yes), n (%)	3373 (6.9)	2708 (5.7)	6081 (6.3)
Psychiatric morbidity^b^ (Yes), n (%)	923 (1.9)	599 (1.3)	1522 (1.6)
**CCI**^**c**^**, n (%)**			
0	46,520 (95.1)	45,320 (95.9)	91,840 (95.5)
1	2119 (4.3)	1764 (3.7)	3883 (4.0)
2 +	274 (0.6)	195 (0.4)	469 (0.5)
**Maternal education, n (%)**			
Lower secondary/primary	5938 (12.7)	2649 (5.7)	8587 (9.2)
Upper secondary	21,764 (46.5)	17,448 (37.3)	39,212 (41.9)
Tertiary	19,129 (40.8)	26,735 (57.1)	45,864 (49.0)
Cohabitating (yes), n (%)	44,827 (91.5)	44,597 (94.2)	89,424 (92.8)
Multiparous (yes), n (%)	26,362 (53.8)	24,534 (51.8)	50,896 (52.8)
All hospital contacts, median (IQI)^d^	3 (1; 9)	3 (1; 7)	3 (1; 8)
In-patient contacts, median (IQI)^d^	0 (0; 1)	0 (0; 0)	0 (0; 0)
ER^e^ contacts, median (IQI)^d^	1 (0; 2)	1 (0; 2)	1 (0; 2)
Out-patient contacts, median (IQI)^d^	2 (0; 6)	1 (0; 5)	2 (0; 5)

^a^As defined in methods section.

^b^Any ICD-10 DF-diagnosis.

^c^Charlson Comorbidity Index.

^d^From index date to end of follow-up. IQI reflects the 25–75-percentile interquartile interval. To comply with Danish data-protection legislation, we report pseudo-medians and pseudo-interquartile intervals, calculated by ordering all observations and averaging the 5 values closest to the relevant percentile.

^e^Emergency room.

In total, 10.3% of responders experienced MHC at baseline, and the HBSC-SCL sum-score differed widely among children experiencing MHC (Supplementary Fig. S1). Children experiencing MHC were more likely to be girls, to have existing psychiatric and somatic morbidity, as well as having a higher CCI score than those who did not (Table 2).

**Table 2:** Characteristics of responders by multiple health complaints (MHC) at the 11-year follow-up of the Danish National Birth Cohort.

	MHC		Total
	No	Yes	
Overall, n (row %)	42,466 (89.7)	4899 (10.3)	47,365 (100.0)
Sex (girls), n (%)	21,510 (50.7)	3240 (66.1)	24,750 (52.3)
Age at index, mean (sd)	11.16 (0.54)	11.17 (0.56)	11.16 (0.54)
Index year, mean (sd)	2011.6 (1.1)	2011.7 (1.1)	2011.6 (1.1)
Somatic morbidity^a^ (Yes), n (%)	2345 (5.5)	363 (7.4)	2708 (5.7)
Psychiatric morbidity^b^ (Yes), n (%)	490 (1.2)	109 (2.2)	599 (1.3)
**CCI**^**c**^**, n (%)**			
0	40,685 (96.0)	4635 (94.9)	45,320 (95.9)
1	1538 (3.6)	226 (4.6)	1764 (3.7)
2 +	171 (0.4)	24 (0.5)	195 (0.4)
**Mother’s educational level, n (%)**			
Lower secondary/primary	2321 (5.5)	328 (6.8)	2649 (5.7)
Upper secondary	15,636 (37.2)	1812 (37.4)	17,448 (37.3)
Tertiary	24,034 (57.2)	2701 (55.8)	26,735 (57.1)
Parents cohabitating (yes), n (%)	40,063 (94.3)	4534 (92.5)	44,597 (94.2)
Multiparous (yes), n (%)	22,022 (51.9)	2512 (51.3)	24,534 (51.8)
All hospital contacts, median (IQI)^d^	3 (1; 7)	4 (1; 12)	3 (1; 7)
In-patient contacts, median (IQI)^d^	0 (0; 0)	0 (0; 1)	0 (0; 0)
ER contacts, median (IQI)^d^	1 (0; 2)	1 (0; 2)	1 (0; 2)
Out-patient contacts, median (IQI)^d^	1 (0; 4)	3 (0; 9)	1 (0; 5)

^a^As defined in methods section.

^b^Any ICD-10 F-diagnosis.

^c^Charlson Comorbidity Index.

^d^From index date to end of follow-up. IQI reflects the 25–75-percentile interquartile interval. To comply with Danish data-protection legislation, we report pseudo-medians and pseudo-interquartile intervals, calculated by ordering all observations and averaging the 5 values closest to the relevant percentile.

### MHC and hospital contacts

**Figure 1:**
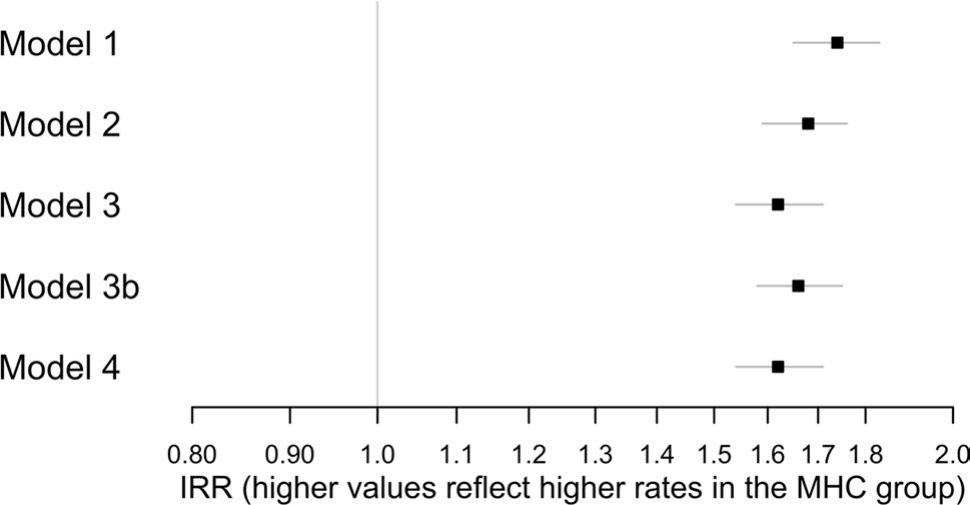
Associations between multiple health complaints (MHC) (yes/no) and number of hospital contacts. Presented as incidence rate ratios (IRR) [95% CI]. Model 1 was unadjusted. Model 2: Model 1 + adjustment for age at index date and sex. Model 3: Model 2 + adjustment for existing somatic and psychiatric morbidity. Model 3b: Model 2 + adjustment for Charlson Comorbidity Index (CCI) score. Model 4: Model 3 + adjustment for maternal parity at the birth of the child, the last completed education by the mother, and parental cohabitation (yes/no) at the child’s index date.

**Figure 2:**
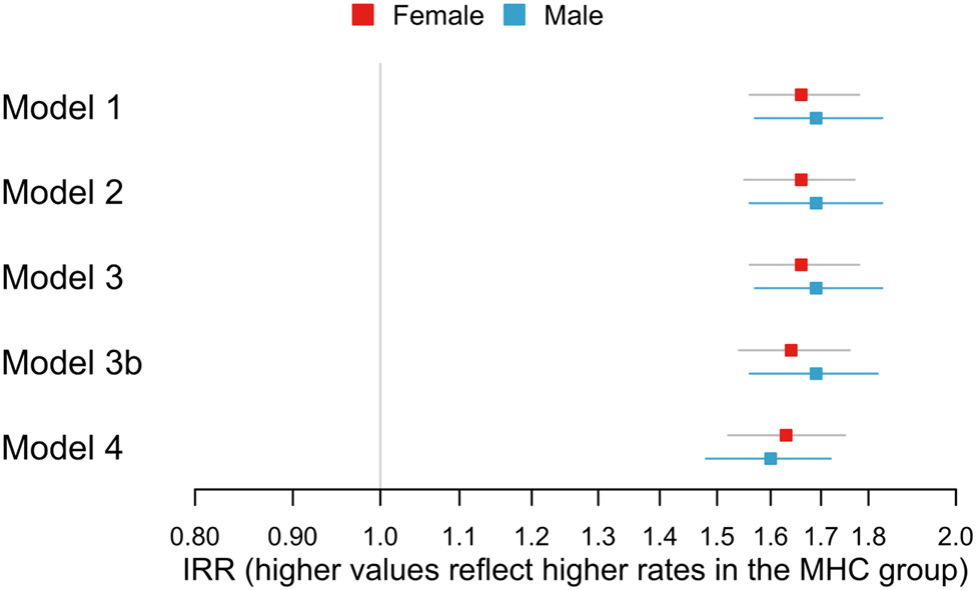
Associations between multiple health complaints (MHC) (yes/no) and number of hospital contacts stratified by sex. Presented as incidence rate ratios (IRR) [95% CI]. Model 1 was unadjusted. Model 2: Model 1 + adjustment for age at index date. Model 3: Model 2 + adjustment for existing somatic and psychiatric morbidity. Model 3b: Model 2 + adjustment for Charlson Comorbidity Index (CCI) score. Model 4: Model 3 + adjustment for maternal parity at the birth of the child, the last completed education by the mother, and parental cohabitation (yes/no) at the child’s index date.

We found unadjusted associations between MHC and total hospital contacts of IRR_Model 1_ = 1.74 [1.65, 1.83] (Fig. 1, Supplementary Table S2). When adjusting for all factors (Model 4), preadolescents experiencing MHC had, on average, 62% more total hospital contacts (IRR_Model 4_ = 1.62 [1.64, 1.72]). In other words, children with MHC were expected to have 0.53 more contacts each year than children without MHC, when holding covariates at reference levels. The association did not appear to attenuate during the 8 years of follow-up (Supplementary Fig. S2) and was similar for each sex (Fig. 2, Supplementary Table S3).

**Figure 3:**
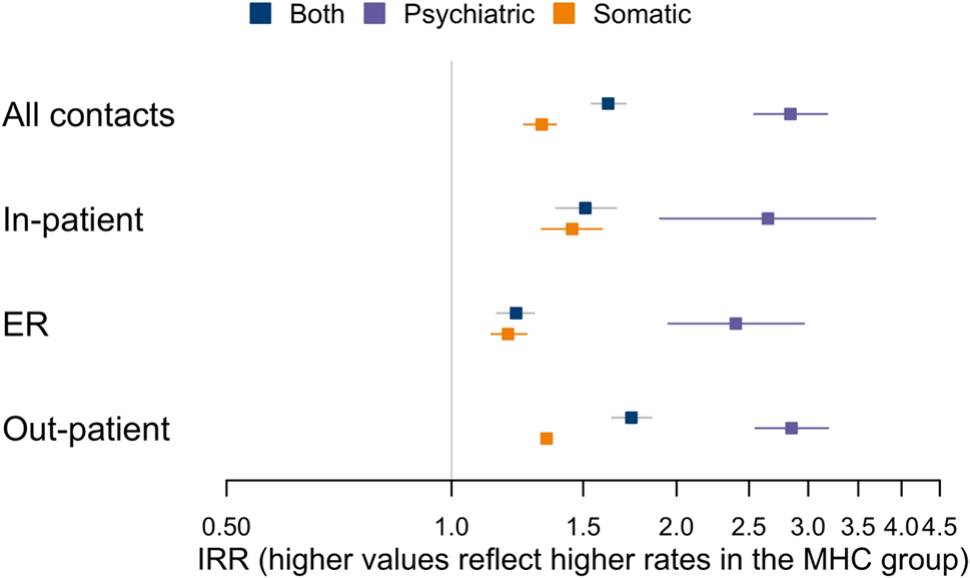
Associations between multiple health complaints (MHC) (yes/no) and number of hospital contacts. Presented as incidence rate ratios (IRR) [95% CI]. Somatic contacts are contacts to any non-psychiatric department. Adjusted for factors in model 4: age at index date, sex, existing somatic and psychiatric morbidity, maternal parity at child’s birth, last completed maternal education, and parental cohabitation at the child’s index date.

**Figure 4:**
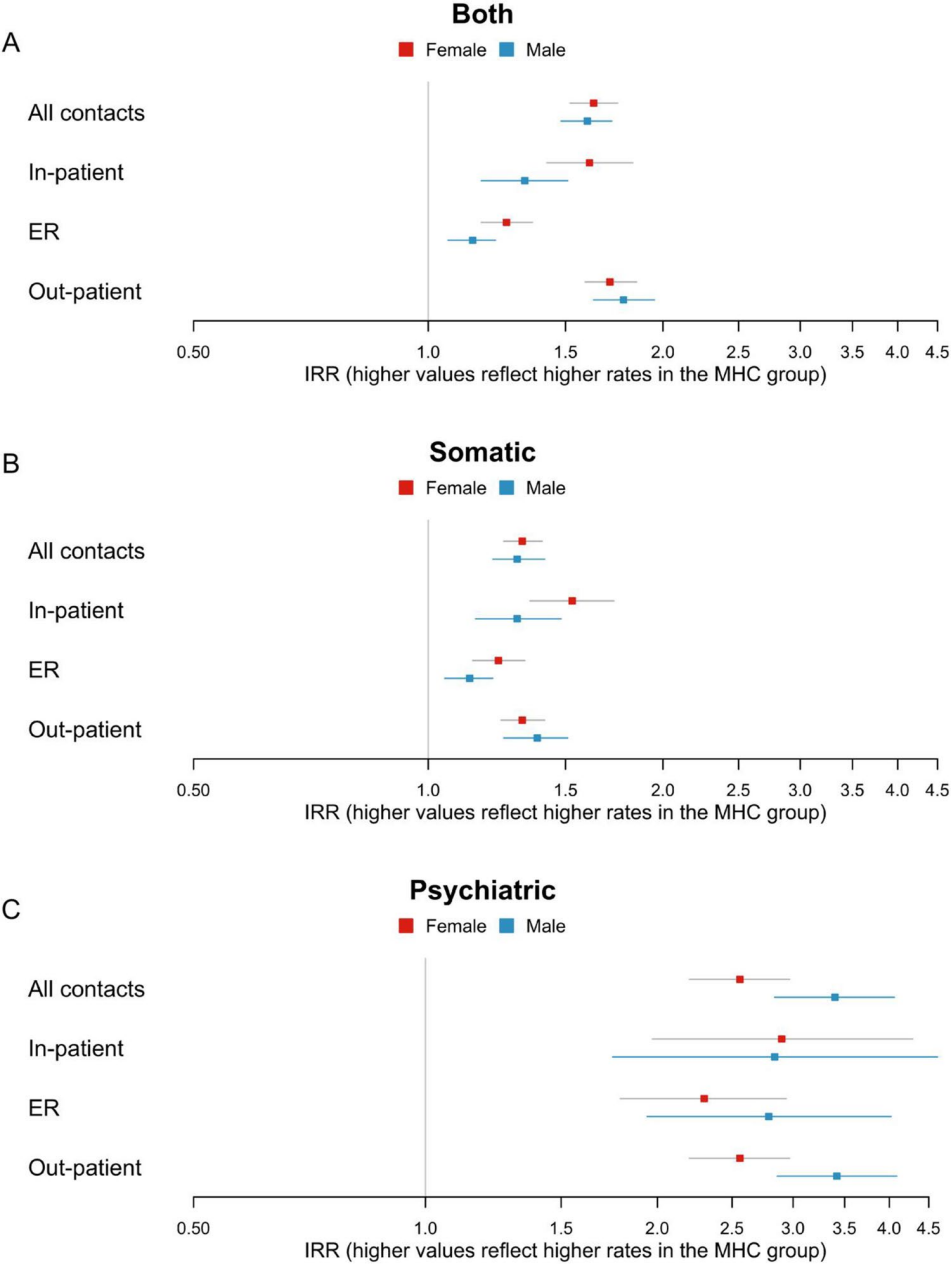
Associations between multiple health complaints (MHC) (yes/no) and number of hospital contacts stratified by sex. Presented as incidence rate ratios (IRR) [95% CI]. Adjusted for factors in model 4: age at index date, sex, existing somatic and psychiatric morbidity, maternal parity at child’s birth, last completed maternal education, and parental cohabitation at the child’s index date.

For estimates including both somatic and psychiatric contacts, the association between MHC and hospital contacts was stronger for in-patient and out-patient contacts than emergency room contacts (IRR_In-patient_ = 1.51 [1.38, 1.66], IRR_Out-patient_ = 1.74 [1.64, 1.85] vs. IRR_Emergency Room_ = 1.22 [1.15, 1.29]). The association was twice as strong for psychiatric contacts (IRR_Psychiatric_ = 2.84 [2.54, 3.18]) as for somatic contacts (IRR_Somatic_ = 1.32 [1.25, 1.38]). When considering emergency, in-patient and out-patient contacts separately, the difference between the somatic and psychiatric departments persisted (Fig. 3, Supplementary Table S4). The contrast was stronger for males, where the association between MHC and somatic contacts was similar to that of females, but the association to psychiatric contacts was roughly 50% higher (Fig. 4, Supplementary Table S5).

### Sum-score and hospital contacts

**Figure 5:**
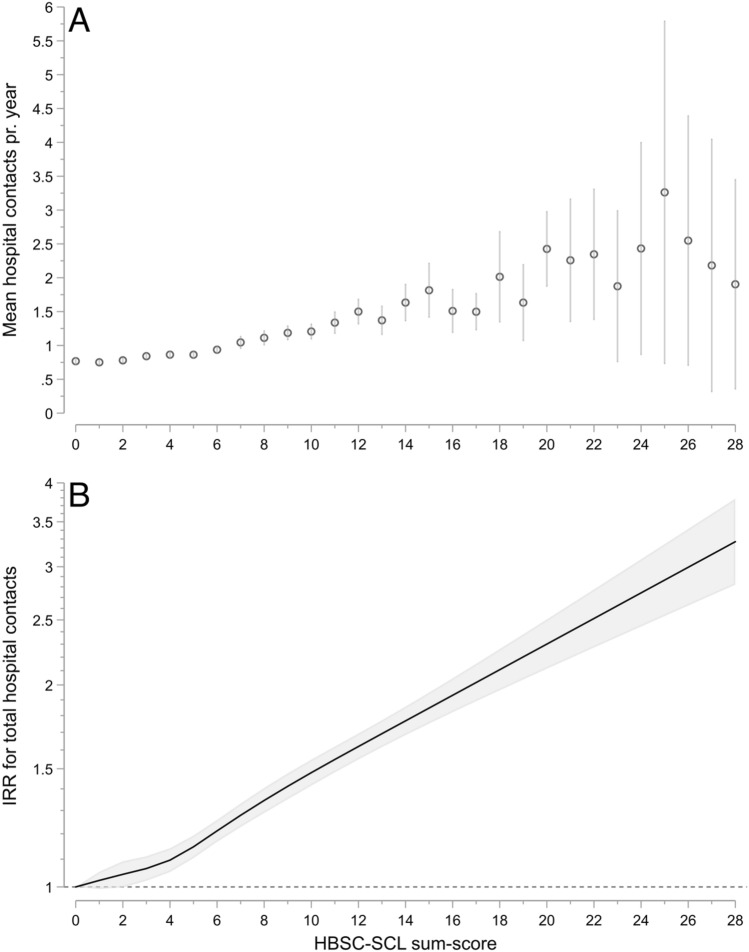
(**A**) Average number of hospital contacts per year as a function of Health Behaviour in School Children Symptom Check-List (HBSC-SCL) sum-score, unadjusted.(**B**) Incidence Rate Ratios (IRR) with 95% CI of total hospital contacts by Health Behaviour in School Children Symptom Checklist (HBSC-SCL) sum-score. Estimated by multivariable negative binomial regression and restricted cubic spline. Adjusted for factors in model 4: age at index date, sex, existing somatic and psychiatric morbidity, maternal parity at child’s birth, last completed maternal education, and parental cohabitation at the child’s index date.

The raw data shows a linear association between the HBSC-SCL sum-score and number of hospital contacts, with hints of a smaller association between sum-score 0–4 but marked associations thereafter (Fig. 5A). These findings were further corroborated by the fully adjusted unrestricted cubic spline regression (Fig. 5B), showing an IRR for hospital contacts of 3.62 [95% CI: 3.12, 4.20] for the maximum sum-score of 28 compared to 0.

## Discussion

In summary, the current study shows that non-specific symptoms in preadolescence are prognostic of hospital contacts during adolescence. This applies whether the symptoms are counted on a discrete scale or dichotomized into MHC. This association was especially strong for psychiatric contacts. While MHC was more prevalent in girls, its associations to hospital contacts were broadly similar between the sexes and it was stable over time. Furthermore, we found a linear relationship between the HBSC-SCL sum-score and total hospital contacts. Taken together, these findings suggest a relevant clinical impact and highlight the urgency in determining the causes of MHC so we can determine which actions to take.

### Context and implications

Previous studies of non-specific symptoms have used a large variety of measures such as the Children’s Somatization Inventory^33,34^, or scales defined specifically for the study in question^8^. Despite this variation, studies consistently find associations between non-specific symptoms and medical help-seeking, both cross-sectionally^33,35^ and prospectively^2^, highlighting that they may be tapping the same construct. For example, in Rytter et al.^2^ we followed the present cohort. After adjusting for sociodemographic variables, responders with both daily somatic and psychiatric symptoms had 50% more contacts to primary care than responders without these symptoms.

The prevalence of MHC was higher among girls, mirroring previous research^1^. However, in previous research, girls not only report more non-specific symptoms, they also appear to feel more impaired^8,33^. Despite this, we found similar associations of MHC to hospital contacts between boys and girls. Whether this is a chance finding is hard to determine as, to the best of our knowledge, no previous research has examined the association between this combination of symptoms and the number of hospital contacts.

The literature on sex as a modifier of the association between a similar construct, self-rated health, and healthcare-seeking is mixed. In a cohort study, Hetlevik et al. followed young adults (aged 25–34 during follow-up) and found modification of sex on number of contacts to general practice^36^. However, our research group found no modification of sex on the association between self-rated health and contacts to general practice in the present cohort^2^.

Compared to previous research, we found an especially strong association for psychiatric contacts. For example, Vila et al. examined the association between "total abdominal pain related impairment" and mental health service use and found no association^33^. However, they examined abdominal pain only. Since MHC is a composite of different symptoms which include psychiatric symptoms, we would expect it to be associated to psychiatric hospital contacts. Also, given that an adolescent must exhibit at least two different symptoms to be labelled with MHC, it should be more closely associated to symptom complexes than phenomena which are entirely monosymptomatic. Some of the explanation may lie in clinical phenomena such as bodily distress or functional somatic symptoms, which are characterized by heterogenous patterns of symptoms that cannot be attributed to verifiable organic pathology^37^.

We also found a linear relationship between HBSC-SCL sum-score and number of hospital contacts. This is in line with the linear relationship found between number of reported symptoms and risk of social disability pension found in 15–26 year olds by Homlong et al.^4^.

Among patients with non-specific symptoms that reach hospital, such as those in the present study, a sub-group will have impairing functional somatic symptoms. These are symptoms that disrupt day-to-day functioning but have no well-defined organic pathology. Græsholt-Knudsen et al.^38^ followed the primary healthcare costs of 5–7 year-olds with impairing functional somatic symptoms for 4.5 years. In line with the increase in number of contacts in the present study, they found that those with impairing functional somatic symptoms cost the healthcare system €178 more per year than those without, even after adjusting for baseline sociodemographic covariates and other child health problems.

As such, our results extend previous findings to non-specific symptoms in the general population by showing their prognostic ability for increased health care use, not only in primary care, but also in the more specialized and expensive healthcare settings of the hospital sector. Importantly, using a measure almost identical to the WHO’s^1^ makes our findings more likely to generalise to the large WHO Health Behaviour in School Children surveys, making them easier to interpret for policymakers.

We argue that this raises a red flag. The WHO measures MHC as an indicator, and this indicator is increasing. If MHC was not associated to healthcare use, it would be easier to interpret MHC as less important from a societal and health care perspective. However, given the clear association between MHC and hospital contacts, and the corresponding healthcare expenditure, we need to understand why this is the case. For the current research, it is still premature to commit to a causal framework for non-specific symptoms. However, causal understanding is required to inform how we best handle the increase, both in society and in the healthcare system. This requires research that can elucidate the causal mechanisms.

### Strengths

The present study is prospective and has up to 8 years of follow-up, eliminating the risk for recall-bias and doubts about whether the reported associations are stable for years.

Furthermore, a large, nationally representative sample increases the precision of our estimates and the generalisability of our results. Since hospitals are only reimbursed for reported contacts, and number of contacts is a largely unambiguous measure, our registry-based outcome data decreases risk of misclassification.

Lastly, the HBSC-SCL is well-validated in the general population and the present age-group.

### Limitations

Some limitations must be kept in mind when interpreting the results. First, at an instrument level, we sought to maintain comparability with the original HBSC-SCL by collapsing two response categories (“more than once a month” and “nearly every month”). It appears not to affect the comparison for MHC, since MHC is dichotomized at a different cut-off, “more than weekly”. It is also unlikely to lead to a poorer estimate of non-specific health symptom frequency, as it has poor discriminatory power at this point^14,15^. However, having the same response categories would have been ideal for generalisability. Furthermore, backache was not included in our scale as we had no information on it. Since backache weekly or more is quite prevalent, ranging from 7.8 to 29%^14,39,40^, we have likely underestimated the prevalence of MHC. Some studies show that the item on backache has appropriate psychometric properties, e.g. that it loads most strongly on the somatic sub-scale^6,16^. Others have found that responses to backache are not invariant^6,14,15^ and that it is the item with the poorest predictive ability^39^. One can argue that the loss of this item in particular may not result in a large loss of information, and thus will not result in a large decrease in precision. However, it may make generalizability less tenable.

Second, the Danish National Birth Cohort has experienced attrition over time with 48% of those invited responding to the 11-year follow-up. The attrition was associated with lower parental education, which in a previous study^2^ was associated with non-specific symptoms. This implies that we may underestimate the true prevalence of non-specific symptoms, as corroborated by us finding a lower prevalence of all individual symptoms than the Danish Health Behaviour in School Children survey^40^ (Supplementary Table S6. Symptom reporting frequencies among all responders.). We also found that non-responders had a slightly higher number of hospital contacts (Table 1). If attrition-rates are particularly high among patients with both MHC and a high number of hospital contacts, this may result in selective response bias, and we may have underestimated the association.

Third, in Denmark, all contacts to hospital are through referral from either a general practitioner or a medical specialist with their own practice. Therefore, number of hospital contacts is likely to be mediated by doctors’ management of non-specific symptoms. As such, our results are more likely to generalize to countries with a similar gatekeeping system, especially if the gatekeepers employ similar management strategies. Furthermore, access to healthcare is free of charge in our sample. For countries where access to healthcare is costly, the pattern of association may be different. Lastly, different cultures interpret symptoms differently^41^; our study is more likely to generalise to populations that have ethic combinations similar to that in Denmark.

### Conclusion

MHC in preadolescents are prognostic of hospital contacts. This shows that we cannot ignore MHC, and to prevent needless healthcare use, we must act. However, to act well, we must understand what to change. Unfortunately, large gaps remain in understanding the causes of MHC, and thus which changes will be most helpful.

## Supplementary Information

Supplementary Information.

## Data Availability

Due to restrictions in Danish law for protecting patient privacy, the dataset used in this study can only be made available through Statistics Denmark. Danish scientific organisations which are university-based can apply for authorization to work with deidentified data within Statistics Denmark, and such organisations can provide access to individual scientists inside and outside of Denmark. Requests for data may be sent to Statistics Denmark: http://www.dst.dk/en/OmDS/organisation/TelefonbogOrg.aspx?kontor=13&tlfbogsort=sektion or the Danish Data Protection Agency: https://www.datatilsynet.dk/english/the-danish-data-protection-agency/contact/.

## Abbreviations

MHC: Multiple health complaints
HBSC-SCL: Health-behaviour in school children-symptom checklist
