# Supplementary material for: Multiple health complaints in preadolescence and hospital contacts during adolescence: a prospective cohort study
Source: Sci Rep. 2022 May 6;12:7412. doi: 10.1038/s41598-022-11167-y (PMC9076908; doi:10.1038/s41598-022-11167-y)
Supplement: Supplementary file 1 — Supplementary Information. [file 41598_2022_11167_MOESM1_ESM.docx]

#

# Supplementary


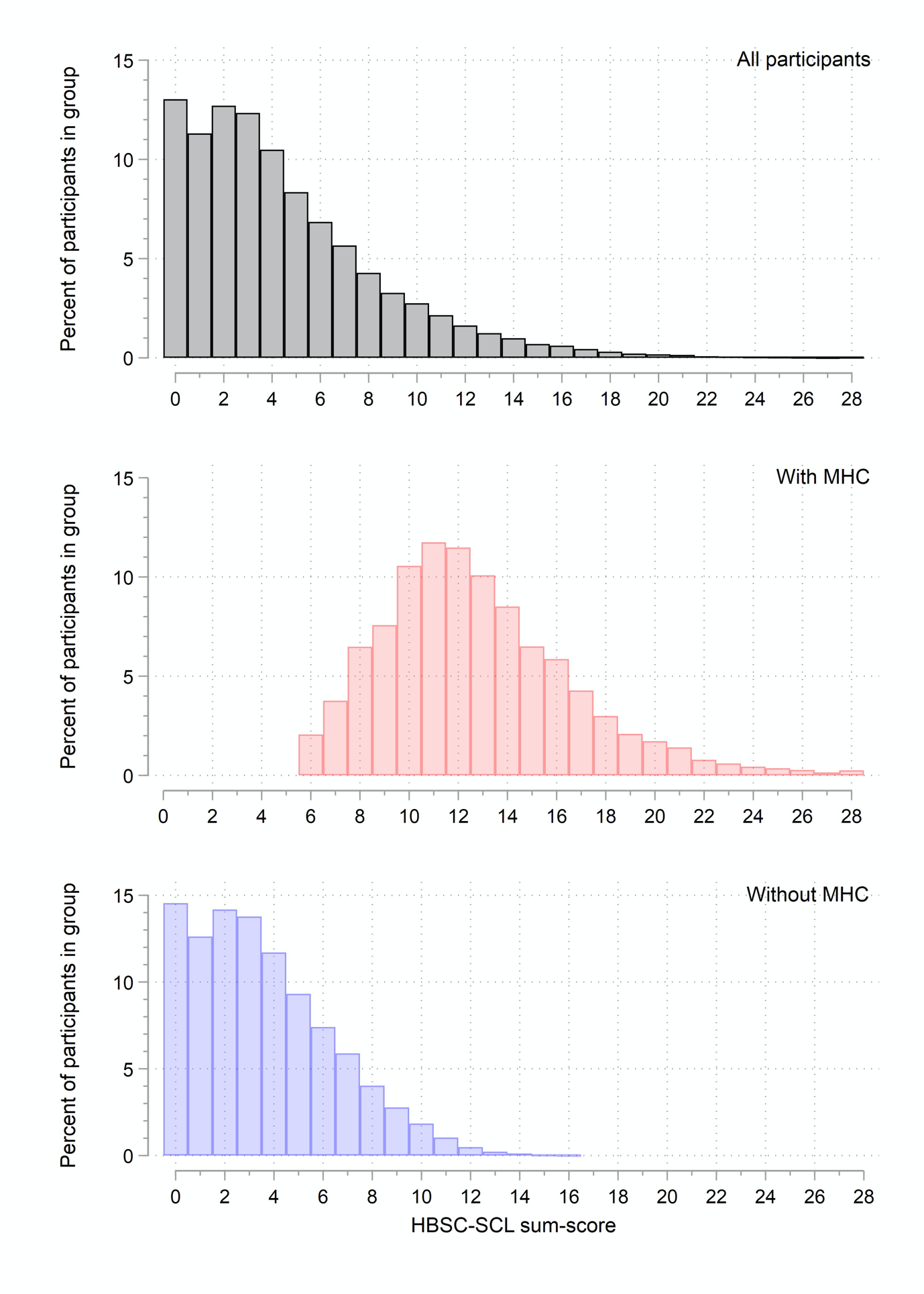


**Supplementary Figure 1:** Frequency of each response category of the Health Behaviour in School Children Symptom Check-List (HBSC-SCL) sum-score among all responders with multiple health complaints (MHC) and responders without MHC. Presented as percentages. Note that the “responders with MHC” distribution’s lower bound is due to its cut-off, ≥2 concurrent symptoms.

**
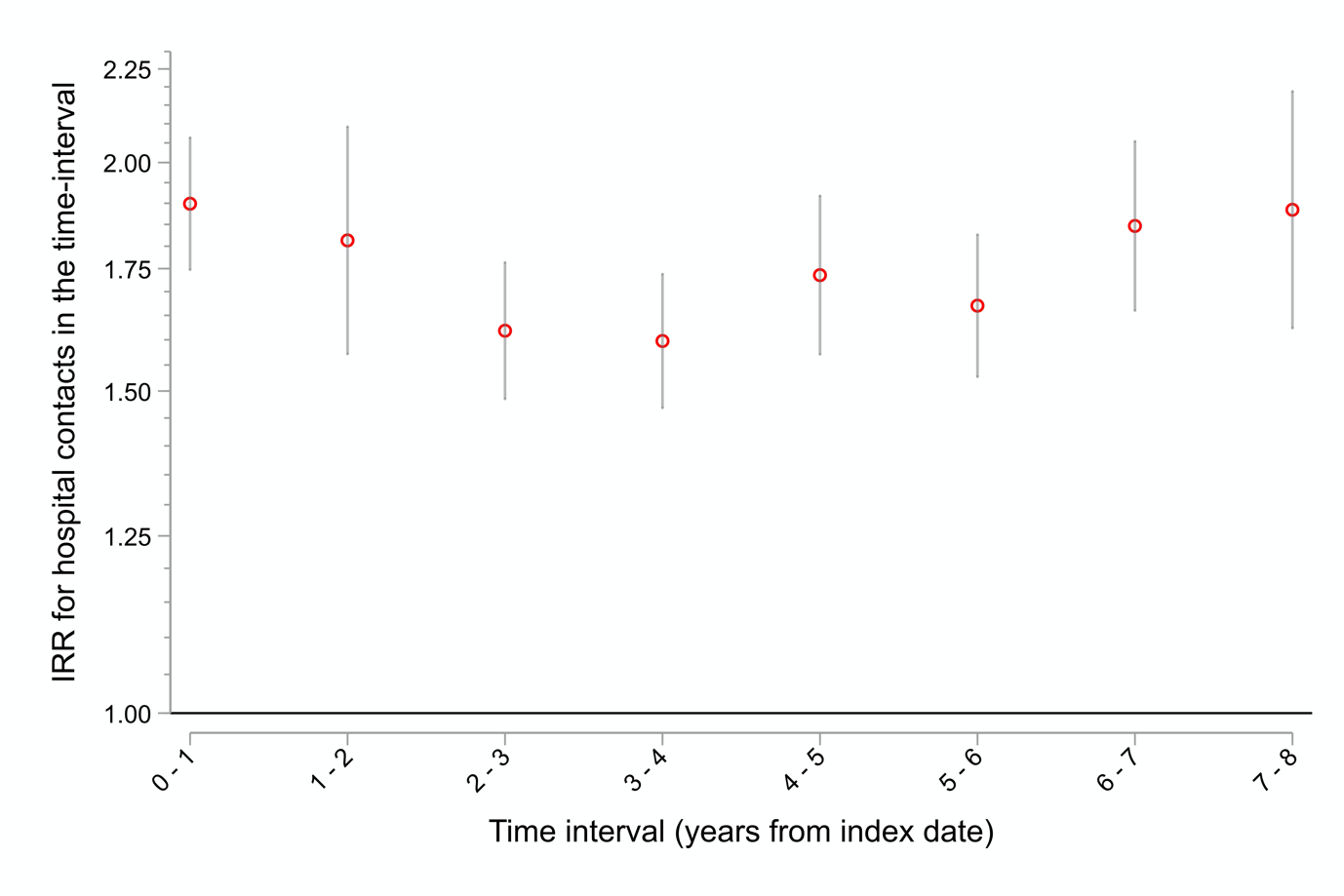
**

**Supplementary Figure 2:** Incidence Rate Ratios (IRR) with 95% CI of total hospital contacts by years from index date. Each estimate is the ratio of contacts in each time interval comparing children with multiple health complaints (MHC) to those without. Estimated by multivariable negative binomial regression and restricted cubic spline. Adjusted for factors in model 4: age at index date, sex, existing somatic and psychiatric morbidity, maternal parity at child’s birth, last completed maternal education, and parental cohabitation at the child’s index date.

.

Supplementary Table 1. ICD-10 Diagnostic Codes for Child Morbidity Prior to Index Date

| Label | ICD-10 code |
| --- | --- |
| Asthma | J45 |
| Arthritis | M08 |
| Diabetes | E10-14 |
| Disease in the nerves, muscles etc. | G71, G80, Q05 |
| Epilepsy | G40 |
| Heart disease | I* |
| Intestinal disease | K50-50, K900 |
| Kidney disease | N0-39 |
| Serious vision or hearing disability | H54, H90-91 |

Supplementary Table 2. Associations between Multiple Health Complaints (MHC) (yes/no) and number of hospital contacts. Presented as incidence rate ratios (IRR) [95% CI].

| IRR [95% CI] for all hospital contacts | | | | | |
| --- | --- | --- | --- | --- | --- |
|  | Model 1 | Model 2 | Model 3 | Model 3b | Model 4 |
| MHC (Yes) | 1.74 [1.65,1.83] | 1.68 [1.59,1.76] | 1.62 [1.54,1.71] | 1.66 [1.58,1.75] | 1.62 [1.54,1.71] |
| Model 1 was unadjusted.  Model 2: Model 1 + adjustment for age at index date and sex. Model 3: Model 2 + adjustment for existing somatic and psychiatric morbidity. Model 3b: Model 2 + adjustment for Charlson Comorbidity Index.  Model 4: Model 3 + adjustment for maternal parity at the birth of the child, the last completed education by the mother, and parental cohabitation (yes/no) at the child’s index date. | | | | | |

Supplementary Table 3. Associations between Multiple Health Complaints (MHC) (yes/no) and number of hospital contacts stratified by sex. Presented as incidence rate ratios (IRR) [95% CI]

|  | | | | | |
| --- | --- | --- | --- | --- | --- |
|  | **Model 1** | **Model 2** | **Model 3** | **Model 3b** | **Model 4** |
| **MHC (Yes), Male** | 1.69 [1.57,1.83] | 1.69 [1.56,1.83] | 1.69 [1.57,1.83] | 1.69 [1.56,1.82] | 1.60 [1.48,1.72] |
| **MHC (Yes), Female** | 1.66 [1.56,1.78] | 1.66 [1.55,1.77] | 1.66 [1.56,1.78] | 1.64 [1.54,1.76] | 1.63 [1.52,1.75] |
| Model 1 was unadjusted.  Model 2: Model 1 + adjustment for age at index date. Model 3: Model 2 + adjustment for existing somatic and psychiatric morbidity.  Model 3b: Model 2 + adjustment for Charlson Comorbidity Index. Model 4: Model 3 + adjustment for maternal parity at the birth of the child, the last completed education by the mother, and parental cohabitation (yes/no) at the child’s index date. | | | | | |

Supplementary Table 4. Associations between Multiple Health Complaints (MHC) (yes/no) and number of hospital contacts. Presented as incidence rate ratios (IRR) [95% CI].

|  | **Somatic** | **Psychiatric** | **Both** |
| --- | --- | --- | --- |
| **All contacts** | 1.32 [1.25,1.38] | 2.84 [2.54,3.18] | 1.62 [1.54,1.71] |
| **In-patient** | 1.45 [1.32,1.59] | 2.65 [1.90,3.69] | 1.51 [1.38,1.66] |
| **Emergency room** | 1.19 [1.13,1.26] | 2.40 [1.95,2.96] | 1.22 [1.15,1.29] |
| **Out-patient** | 1.34 [1.27,1.42] | 2.85 [2.55,3.19] | 1.74 [1.64,1.85] |

Somatic contacts are contacts to any non-psychiatric department. Adjusted for factors in model 4: age at index date, sex, existing somatic and psychiatric morbidity, maternal parity at child’s birth, last completed maternal education, and parental cohabitation at the child’s index date.

Supplementary Table 5. Associations between Multiple Health Complaints (MHC) (yes/no) and number of hospital contacts stratified by sex. Presented as incidence rate ratios (IRR) [95% CI].

|  | | | | | | | | | | | |
| --- | --- | --- | --- | --- | --- | --- | --- | --- | --- | --- | --- |
|  | **Somatic** | | **Psychiatric** | | | **Both** | | | | | |
|  | **Male** | **Female** | **Male** | **Female** | | **Male** | **Female** | | | | |
| **All contacts** | 1.30 [1.21,1.41] | 1.32 [1.25,1.40] | 3.40 [2.84,4.06] | | 2.56 [2.20,2.97] | 1.60 [1.48,1.72] | | | | | 1.63 [1.52,1.75] |
| **In-patient** | 1.30 [1.15,1.48] | 1.53 [1.35,1.73] | 2.84 [1.75,4.62] | | 2.90 [1.97,4.29] | 1.33 [1.17,1.51] | | | | 1.61 [1.42,1.83] | |
| **Emergency room** | 1.13 [1.05,1.21] | 1.23 [1.14,1.33] | 2.79 [1.94,4.02] | | 2.30 [1.79,2.94] | 1.14 [1.06,1.22] | | | 1.26 [1.17,1.36] | | |
| **Out-patient** | 1.38 [1.25,1.51] | 1.32 [1.24,1.41] | 3.42 [2.86,4.09] | | 2.56 [2.20,2.97] | 1.78 [1.63,1.95] | | 1.71 [1.59,1.85] | | | |

Adjusted for factors in model 4: age at index date, sex, existing somatic and psychiatric morbidity, maternal parity at child’s birth, last completed maternal education, and parental cohabitation at the child’s index date. Somatic contacts are contacts to any non-psychiatric department.

Supplementary Table 6. Symptom reporting frequencies among all responders.

|  | **Rarely or never** | **Nearly every month** | **About every week** | **More than once a week** | **Nearly every day** |
| --- | --- | --- | --- | --- | --- |
| **Headache** | 64.2% | 24.4% | 5.4% | 4.4% | 1.6% |
| **Stomach ache** | 63.9% | 27.5% | 4.4% | 2.8% | 1.3% |
| **Feeling low** | 44.0% | 40.8% | 9.0% | 4.8% | 1.4% |
| **Irritable** | 35.0% | 44.3% | 12.0% | 7.0% | 1.7% |
| **Nervous** | 55.1% | 34.6% | 6.5% | 3.0% | 0.9% |
| **Difficulty falling asleep** | 52.3% | 25.3% | 8.5% | 7.2% | 6.8% |
| **Dizziness** | 82.7% | 13.6% | 1.9% | 1.3% | 0.5% |
